# Physiotherapy in an advanced rehabilitation pathway for patients after hip and knee arthroplasty: A proposal

**DOI:** 10.4102/sajp.v77i1.1565

**Published:** 2021-09-28

**Authors:** Retha-Mari Prinsloo, Monique M. Keller

**Affiliations:** 1 Department of Physiotherapy, School of Therapeutic Sciences, University of the Witwatersrand, Parktown, South Africa

**Keywords:** length of stay, early mobilisation, physiotherapy, arthroplasty, replacement, hip, knee

## Abstract

**Background:**

Accelerated rehabilitation pathway (ARP) decrease patients’ hospital length of stay (LOS). A lack of evidence exists on physiotherapy management and outcome as part of ARP in South Africa (SA). Our study will aim to determine whether early mobilisation and increased frequency of physiotherapy treatments for participants after hip or knee arthroplasty surgery on post-operative day 0 (POD 0) affect outcome.

**Methods/design:**

A quantitative prospective cohort study incorporating ARP on (*n* = 60) non-randomised elective hip and knee arthroplasty participants will be compared with a more conservatively managed historical control group (*n* = 60). The physiotherapy protocol includes early mobilisation and exercises 1–3 h post-operatively on POD 0 and a second mobilisation and exercise session, 1–2 h later. Outcomes measures are as follows: hours for LOS, the WOMAC measured pre-operatively, 6 weeks and 3 months post-operatively, 30-day readmission for safety and cost comparison between the prospective and historical cohorts. Descriptive statistics will be undertaken. A paired *t*-test will be used to analyse each of the outcome measures across the time periods if data are normally distributed. Length of stay, WOMAC score and cost data will be compared between the groups, using a Mann–Whitney U test. The occurrence of adverse events will be compared between the groups using Pearson’s chi-square tests. The confidence interval will be set at 95% and *p* = 0.05 will be considered statistically significant.

**Discussion:**

Globally, ARP’s are successfully implemented to manage patients presenting with hip and knee osteoarthritis (OA). Research investigating physiotherapy protocols in an ARP is lacking in the literature.

**Conclusion:**

Achieving the same-day discharge after hip and knee arthroplasty surgeries may help elective surgery backlogs and waiting lists in a more cost-effective manner.

**Clinical implications:**

The same day discharge after arthroplasty may be a cost-effective management option in the future.

**Protocol identification:**

Pan African Clinical Trial Registry, PACTR202103637993156.

## Introduction

Increased life expectancy, lifestyle changes, obesity and non-communicable diseases, trauma and HIV contribute to increased musculoskeletal and orthopaedic diseases such as osteoarthritis (OA) in South Africa (SA) (Plenge et al. 2018). OA is one of the leading causes of pain in patients’ hips and knees and chronic disability, with a resulting adverse effect on quality of life and function (Lespasio et al. 2017). Hip and knee arthroplasty surgeries are very effective in treating end-stage OA (Sculco & Pagnano 2015).

Kurtz et al. (2007) determined that in the United States of America (USA) the demand for total hip arthroplasties would increase by 174% and for total knee arthroplasties by 673% by 2030 from the demand seen in 2007. The long-term effect is an increased demand for hip and knee arthroplasty procedures enabling individuals to live a fully functional life. It, however, places a directly increased financial strain on private and public stakeholders and medical funders. With National Health Insurance (NHI) on our doorstep in South Africa, the cost of this increased demand will be substantial for the SA healthcare system and medical funders. Decreasing hospital length of stay (LOS) after elective total hip and knee arthroplasty surgical procedures may impact the individual, medical funders and government stakeholders positively. In addition, the coronavirus disease 2019 (COVID-19) pandemic has negatively affected waiting times for surgery, leaving a backlog of patients waiting for elective hip and knee arthroplasty surgeries. Over the last year and a half many countries decided to cancel elective surgeries to reserve resources and personnel to prioritise management of patients infected with COVID-19 (Lancet 2021).

When considering the increased cost and burden on resources, there has been a national and international shift towards advanced multidisciplinary approaches for hip and knee arthroplasty surgeries. A variety of names are used in the literature for these advanced protocols. Advanced clinical pathway (Plenge et al. 2018), fast track (Tayrose et al. 2013), accelerated rehabilitation protocol (Robertson et al. 2015) and rapid recovery are drawing more and more attention worldwide for an alternative method of management and rehabilitation of patient undergoing total hip and knee arthroplasty surgeries.

Studies conducted on a fast track or ARP demonstrate that advanced protocols can be implemented safely, effectively and reduce the hospital’s LOS, without increasing complications more than conservative protocols (Lazic et al. 2018; Riemer et al. 2017; Robertson et al. 2015). Evidence of the implementation of advanced clinical pathways and enhanced recovery after surgery (ERAS) in a SA context shows that advanced clinical pathways can be implemented safely and reduce LOS (Immelman et al. 2018; Riemer et al. 2017). As a result of the decreased LOS, the healthcare system’s cost-saving is of great value moving towards the NHI and considering the aftermath of the Covid-19 pandemic.

Although advanced pathways have different names, the focus of these protocols is standardised care. The standardised care includes patient education, pain control, thromboprophylaxis (preventing blood clot formation in blood vessels), managing blood loss during surgery and early mobilisation, as the key elements (Lazic et al. 2018; Riemer et al. 2017).

We will now describe the specific core adaptations included in advanced protocols. The pain control is a focussed multi-modal, opioid sparing regime to decrease nausea, dizziness and sleepiness after surgery, thus enabling patients to mobilise within hours of the surgery. Intermittent pneumatic compression pumps (IPCPs) are used post-operatively for thromboprophylaxis. Depending on the patient’s risk profile, aspirin or clexane is prescribed. Blood loss in surgery is restricted by controlled hypotension, no drainage pipes, using a tourniquet at appropriate pressures only during the surgery’s cementation and tranexamic acid. The surgical technique is a minimally invasive, muscle-sparing approach with kinematic alignment and subcutaneous sutures with tissue adhesives that contribute to rapid recovery (Lazic et al. 2018).

The advancement in elective total hip and knee arthroplasty has reached a point where decreasing the LOS by using advanced pathways led to outpatient joint arthroplasties in the United States of America and Europe. In 2018, total knee arthroplasties were no longer only in-hospital procedures. According to Yates et al. (2018), more than half of the American Academy of Hip and Knee Surgeons were instructed by their hospitals to implement outpatient protocols. One day or outpatient arthroplasty, according to Sculco and Pagnano (2015) is the way forward. In outpatient or 1-day total hip and knee arthroplasty surgeries, patients are discharged on the same day as the surgery. Outpatient total joint arthroplasties can be implemented safely, cost-effectively and are beneficial to patients. Increased LOS is associated with higher morbidity and mortality (Lazic et al. 2018; McCulloch et al. 2017). In SA, outpatient arthroplasty surgeries have not yet been performed.

Physiotherapy forms an integral part of the advanced pathways through patient education, management before the surgery, early mobilisation and rehabilitation. Early mobilisation (patient ambulating or walking with an appropriate mobility aid away from the bed), plays an essential role in decreasing post-operative complications, including deep vein thrombosis (DVT), prosthesis-related infections and postural hypotension (Chen et al. 2012; Dossett & Chesser 2017). Physiotherapy intervention also improves patients’ function and muscle strength (Chen et al. 2012; McCulloch et al. 2017). When considering outcomes after total knee arthroplasty, Kolisek et al. (2009) reported that joint range of motion (ROM) and function were comparable with the final follow-up for both patients having surgery out- and in-hospital. In total hip arthroplasty surgeries, the pain and Harris Hip score were similar 4 weeks post-operatively (Goyal et al. 2016).

Studies have shown that early mobilisation post-operative day 0 (POD 0) plays a significant role in decreased LOS (Lazic et al. 2018; Masaracchio et al. 2017; McCulloch et al. 2017; Riemer et al. 2017; Tayrose et al. 2013; Yakkanti et al. 2019). In a systematic review and meta-analysis of 17 studies, Masaracchio et al. (2017) found that early initiation of rehabilitation on POD 0 decreases the LOS without increasing adverse events in patients following joint replacement surgery. Tayrose et al. (2013) reported that patients mobilised in the recovery room directly following the surgery, while others reported mobilising patients 2–4 h POD 0 (Raphael et al. 2011).

Despite the advancements in decreased LOS in the hospital for patients who underwent hip and knee arthroplasty surgeries, studies indicate a 10% and 20% patient dissatisfaction percentage for outcomes (Gunaratne et al. 2017). Patient satisfaction depends on factors such as residual pain, stiffness, post-operative function, post-operative complications and pre-operative patient expectations (Walker et al. 2018). Patient expectations are usually based on improved function, decreased pain, decreased stiffness and an overall increase in quality of life (Gunaratne et al. 2017; Thambiah et al. 2015).

The orthopaedic surgeon at the private hospital where our study is to be carried out has implemented an ARP and early POD 0 mobilisation. For the ARP, the average LOS has decreased from 3.5 to 2.4 days and now is 23 h. When talking about day-surgery, McCulloch et al. (2017) made a vital point about the LOS documentation after hip and knee arthroplasty surgeries. Length of stay measurement should be performed in hours and not in days to be more precise in documenting the time taken before discharge. Patients who have their operation later in the day may be discharged after a similar time as the patient who was operated on first. Length of stay measured in hours prevents confusion and improves accurate documentation of the actual time from going to the operating theatre, till the time of discharge.

If early mobilisation (patient ambulating or walking with an appropriate mobility aid away from the bed) and increased frequency of physiotherapy intervention on POD 0 lead to decreased LOS, stakeholders and medical funders will benefit from the cost-saving reduced length of hospital stay in the private and public sectors in SA. Private and public healthcare, the SA government and NHI can accommodate the increased demand for joint arthroplasties by effectively using the available resources and saving on costs. Patients may benefit from a shorter waiting period for their hip or knee arthroplasty leading to a quicker recovery and better quality of life. Good quality healthcare must be provided cost-effectively and improve patient-reported outcomes and satisfaction. The ARP provides a possible improved way of elective total hip and knee arthroplasties.

Appropriate physiotherapy management plays an integral part in the ARP. With a lack of evidence guiding clinical practice in SA, our study thus aims to determine how early mobilisation and increased daily frequency of physiotherapy on POD 0 impact the hospital LOS, safety and patient satisfaction, after hip and knee arthroplasty in a private hospital in SA.

The specific objectives of our study are to:

- determine how early mobilisation and the increased frequency of physiotherapy on POD 0 impacts hospital LOS in mean hours, (primary outcome)
- determine patient satisfaction (pain, function, stiffness and expectation) 6 weeks and 3 months post-operatively
- evaluate the safety of implementing an accelerated rehabilitation pathway on patients after hip and knee arthroplasty, documenting any adverse events and 30-day readmission rate
- determine and compare the costs of LOS, in a simple cost comparison between historic cohort and prospective cohort groups.

## Method

Our study is a prospective cohort study that includes a purposive convenient, selected sample of patients (*n* = 60). The ARP guides the management after total hip and knee arthroplasty surgery. The prospective cohort will be compared with a historical control group (*n* = 60) managed with an older protocol. The sample was determined by the total number of patients who underwent total hip and knee arthroplasties in the historical control group at the private hospital performed by the same orthopaedic surgeon as for the prospective group. The setting and multidisciplinary team for both the prospective participants and the historical control group is the same.

All consecutive elective hip and knee arthroplasty patients cleared pre-surgically by the physician as per ARP will be included. Patients with revision surgery, trauma-related surgeries, bilateral arthroplasty, poor balance and cognitive deficiencies will be excluded from participation.

All patients in both the historical and prospective groups received the same treatment and protocol at the Medicare private hospital in Rustenburg, the difference lying in the time before first mobilisation and the frequency of treatment on POD 0. The historical group received a more conservative protocol that included an educational session in hospital pre-operatively and mobilisation once on POD0-3 hours post-operatively.

The prospective group will receive the new protocol, that is, an educational session the week prior to surgery and before hospital admission; mobilisation 1–3 h post-operatively (Raphael et al. 2011; Tayrose et al. 2013) and then again for a second time 1–2 h thereafter.

Information will be provided to all potential participants and informed consent to be included in our study will be obtained. The multidisciplinary team for all surgeries consists of the same group of individuals: orthopaedic surgeons, anaesthetists, physiotherapists and nursing staff. The protocols will be uniform throughout our study except for the differences described here.

### Pre-intervention protocol and assessment for hip and knee arthroplasty

The physiotherapy protocol will start with pre-operative education, pre-habilitation (rehabilitation performed by the physiotherapist before the surgery) and include an evaluation session (measurement of hip and knee range of movement (ROM) with a goniometer, hip and knee muscle strength with the Oxford Scale and The Western Ontario and McMaster Universities Osteoarthritis Index (WOMAC) questionnaire is also completed in this session). This session emphasises post-operative expectations for the patient and physiotherapist, post-operative exercises, bed mobility, gait re-education, navigating stairs with crutches, preventive measures, early mobilisation and ice programme (Andersen et al. 2021). The exercises consist of foot pumps to encourage circulation, heel slides exercises to improve hip and knee ROM, static quadriceps muscle strengthening exercises to improve knee support, free active hip abduction ROM/functional exercises and to encourage joint ROM. The patients are instructed to do one set of 10 repetitions, a minimum of five times per day. The education session also includes discharge requirements, information about in-hospital and follow-up physiotherapy sessions, education regarding the home environment, safety aspects at home and preparing the patient for a possible same day discharge (Andersen et al. 2021; Thompson et al. 2021). Preventive hip arthroplasty measures implemented for the first 3 months post-operatively include no hip internal rotation, no hip adduction (crossing the legs) and no hip flexion more than 90°. The patient is provided with a printed information sheet with all the exercises for pre- and post-operative management. The same exercises are used for both pre-habilitation and post-operatively. The physiotherapy protocol used in the ARP will now be explained in detail.

### Protocol after returning from the recovery room

Post-operatively, patients are routinely monitored in the ward by the nursing staff for nausea, hypotension, tachycardia or desaturation. An ice pack is applied as soon as the patient is back in the ward after surgery. Ice is applied almost continuously during the day through a light compression bandage (Tubigrip). Patients are advised to apply the ice for 8 h per day with short intervals without ice for the first 3 days. Karaduman et al. (2019) also advocated using ice for a total of 8 h per day, icing for 2 h periods four times per day, in the acute phase after arthroplasty.

Patients have no catheter or drainage pipes. Patients return to the ward with a short drip in the arm and cardiac monitor electrodes on their chests, which is removed to make mobilisation easier. An intermittent pneumatic compression pump is in place around the patient’s calves to help in preventing DVTs. Patients are mobilised POD 0 as soon as they are fully awake, no nausea or dizziness is observed and with the surgeon’s permission, 1–3 h post-operatively (Raphael et al. 2011; Tayrose et al. 2013; Yakkanti et al. 2019). Patients are encouraged to start eating and drinking as soon as possible. The physiotherapy post-operatively starts by making the patient sit more upright (while respecting hip flexion precaution of 90° after total hip replacements) to assist with hypotension. Bed exercises consisting of foot pumps, static quadriceps muscle strengthening exercises, free active hip abduction ROM/functional exercises and heel slides exercises to encourage joint ROM are performed (Jenkins et al. 2019; Thompson et al. 2021).

The bed exercise prescription is one set of 10 repetitions, a minimum of five times per day. The patient is then mobilised to sit over the side of the bed. While the patient sits over the side of the bed, a short lever quadriceps extension exercise is prescribed. If the patient has no dizziness, nausea or intense pain and adequate knee proprioception (Jenkins et al. 2019) in the operated knee, standing with a walking frame follows. Gait and knee-locking are explained and a revision of the education that was performed during the pre-habilitation phase is undertaken. Then, the patient mobilises from the bed to the toilet and back, approximately 20 m in total. If the patient is mobilising well with a walking frame, he or she can walk with elbow crutches. Ice is re-applied after the mobilisation. The patient is encouraged to sit out in a chair for lunch and dinner.

A second physiotherapy session, 1–2 h after the first physiotherapy session, follows for the prospective group. The second physiotherapy session comprises the same exercises and mobilisation programme as the first session. The physiotherapist will also help the patient to get dressed in their clothes during the second session. In-between the two physiotherapy sessions, the patient, may mobilise with the nursing staff’s assistance to the bathroom if the need arises. Depending on how well and safe the patient is in mobilising, stair climbing might be included in the second session on POD 0 or in the first physiotherapy session on post-operative day 1. Patients will receive physiotherapy sessions twice per day while in hospital.

### Post-intervention assessment and protocol before discharge

Before discharge, the patient is expected to demonstrate good independent bed mobility (be able to get in and out of bed by themselves), mobilise 50 m or more with an appropriate assistive device, navigate by climbing five stairs safely with crutches or one step in case of a walking frame (Berger et al. 2009; Thompson et al. 2021). Range of motion should be close to 90° knee flexion and 90° hip flexion for knee and hip arthroplasty. When the patient is medically stable, pain is under control (patient indicating that they feel they will manage at home using pain tablets with a visual analogue scale rating of equal or less than 5 out of a maximum of 10 pain), and the physiotherapy criteria are met, the patient is discharged home (Thompson et al. 2021). A home exercise programme that was already provided pre-operatively is continued. The home exercise programme after a knee arthroplasty includes foot pumps (ankle dorsiflexion and plantar flexion), knee extension static quadriceps, knee flexion and extension heel slides, knee extension over a foam roller or pillow, hip flexion straight leg raise, knee extensions through full ROM while seated. The exercise prescription for all the exercises is one set, 10 repetitions, five times per day with progression during follow-up physiotherapy outpatient visits. The home exercise programme after a hip arthroplasty includes foot pumps (ankle dorsiflexion and plantar flexion), knee extension static quadriceps, knee flexion and extension heel slides within limits, hip abduction within limits of pain, knee extensions through full ROM while seated. In standing knee flexion hamstring curls, hip abduction and hip extension exercises are added. The exercise prescription for all the exercises is one set, 10 repetitions, five times per day with progression during follow-up physiotherapy outpatient visits. A follow-up appointment for outpatient physiotherapy is arranged. Patients will follow-up once per week for 6 weeks. The WOMAC questionnaire will be completed again at 6 weeks and 3 months after surgery.

Upon completing the prospective cohort study, a retrospective comparison with data from our historical control will be conducted including a simple cost comparison.

### Outcome measures

Length of stay is frequently used as an outcome measure after hip and knee arthroplasty surgeries and will be measured in hours to be more precise and to be able to detect more effectively any small changes in the time period. Length of stay will be measured in hours from when the patient goes to the theatre to when the patient is discharged to be accurate (McCulloch et al. 2017).

The WOMAC, a patient-administered questionnaire, is a widely used, valid and reliable outcome measure in patients with hip and knee arthroplasty. It measures pain, stiffness and physical function (Collins et al. 2011; Giesinger et al. 2015). Cronbach’s coefficient alpha scores are 0.86 for pain, 0.90 for stiffness and 0.95 for physical function. The reliability intraclass correlation coefficients scores are high measured at 0.88 for pain, 0.76 for stiffness and 0.91 for physical function (Bellamy et al. 1988; Söderman & Malchau 2000). The WOMAC score is rated on an ordinal scale of 0 to 4. Sub-scores or global scores are calculated, with lower scores indicating lower levels of pain, stiffness and physical disability. Data collected from the WOMAC will be analysed against patient satisfaction measured on a 5-point Likert scale (extremely satisfied, satisfied, neutral, dissatisfied and extremely dissatisfied). According to answers, there will be two groups, satisfied (extremely satisfied and satisfied) and unsatisfied (neutral, dissatisfied and extremely dissatisfied) (Thambiah et al. 2015). The WOMAC questionnaire will be administered pre-operatively and repeated at 6 weeks and 3 months post-operatively. Walker et al. (2018), identified values for the total WOMAC scores that are predictive of patients’ level of satisfaction.

The proposed protocol’s safety will be measured with the 30-day readmission rate. Unplanned readmissions within 30 days after the patient is discharged from the hospital will be documented (Rumball-Smith & Hider 2009). Finally, the direct cost of LOS will be compared in a mini cost analysis between the prospective and historical cohorts by calculating the cost of direct hospital expenses incurred per day, theatre time, assistive devices (crutches or walking frames), prosthetics, physiotherapy fees and orthopaedic specialist fees.

### Data management

All data obtained will be safely kept electronically on the first author’s password-protected computer for 6 years if not published and 2 years if published. The informed consent and questionnaire hard copies will also be stored securely in the first author’s code-protected office for 6 years.

### Statistical analysis

Descriptive statistics, namely frequencies and percentages for categorical data and mean and standard deviation or median and percentiles for numerical data, will be calculated. Quantitative outcome variables will be tested for normality using the Shapiro–Wilk test. International Business Machines Statistical product and service solutions (IBM SPSS) version 27 will be used to analyse the data. A paired *t*-test will be used to analyse each of the outcome measures across the time periods if data are normally distributed. Length of stay, WOMAC score and cost data will be summarised using median and inter-quartile ranges and compared between the two groups, using a non-parametric Mann–Whitney U test. The occurrence of adverse events will be compared between the two groups using Pearson’s chi-square tests. Sub-group analysis will be performed based on age, gender and type of arthroplasty performed. The confidence interval will be set at 95% and power of probability of *p* = 0.05 will be considered statistically significant.

### Validity and reliability

Validity will be ensured by using consistent, standardised verbal instructions during the protocol. Reliability will be improved by collecting the data in a standardised environment and implementing standardised procedures.

### Ethical considerations

The trial is registered with the Pan African Clinical Trial Registry (PACTR202103637993156). Ethical clearance was obtained from the University of the Witwatersrand Human Research Ethics (Medical) Committee (reference number: M200576), the orthopaedic surgeon and manager of the private hospital in Rustenburg. Permission for data from hospital records for the data collection in the main prospective and historical cohort group will be obtained. Information will be given to prospective participants and written permission to participate in our study will be requested.

## Discussion

Hip and knee pain because of osteoarthritis is one of the leading causes of pain, disability and decreased life quality. Hip and knee arthroplasties have become the answer for optimal function and quality of life. Using an ARP pathway in hip and knee arthroplasty surgeries on patients waiting for elective surgeries may decrease the already long waiting lists (Wainwright 2021) in the public sector and address the backlog in the private sector in SA.

Accelerated rehabilitation protocol has become very popular internationally and is gaining popularity in SA (Immelman et al. 2018; Riemer et al. 2017). A multidisciplinary team in Rustenburg, SA, has started implementing an ARP affecting LOS, with the first same-day discharge from the hospital. The costs saved because of a decrease in LOS may be beneficial for medical funders, stakeholders such as the government as they plan to implement the NHI in the future.

There is a lack of evidence of the physiotherapy protocol in the ARP (Anderson et al. 2021). Thus, the proposed study investigates the physiotherapy protocol on how early mobilisation and increased frequency of physiotherapy treatments, that is, twice per day, for participants after hip or knee arthroplasty surgery on POD 0 in a private hospital in SA affects outcomes. The outcomes include LOS in hours and not days, patient satisfaction (pain, function, stiffness and expectation) with the WOMAC, safety by documenting any readmission within 30 days of the hip and knee arthroplasty, with an added simple cost comparison between the prospective and historical cohorts to indicate any cost-savings if present.

## Conclusion

Our study will determine the LOS, patient satisfaction, safety and cost comparison of early mobilisation and frequency of physiotherapy compared with a historic physiotherapy protocol. The feasibility of a physiotherapy protocol in an ARP will thus be evaluated and may provide a cost-effective rehabilitation method in a resource restraint SA.
